# Peculiar macrophagous adaptations in a new Cretaceous pliosaurid

**DOI:** 10.1098/rsos.150552

**Published:** 2015-12-23

**Authors:** Valentin Fischer, Maxim S. Arkhangelsky, Ilya M. Stenshin, Gleb N. Uspensky, Nikolay G. Zverkov, Roger B. J. Benson

**Affiliations:** 1 Department of Earth Sciences, University of Oxford, Oxford OX1 3AN, UK; 2 Department of Geology, Université de Liège, 14 allée du 6 Août, Liège 4000, Belgium; 3 Ecological Faculty, Saratov State Technical University, Politekhnicheskaya Ul. 77, Saratov 410054, Russia; 4 Geological Faculty, Saratov State University, Astrakhanskaya Ul. 83, Saratov 410012, Russia; 5 I.A. Goncharov Ulyanovsk Regional Natural History Museum, Boulevard Novyi Venets 3/4, Ulyanovsk 432000, Russia; 6 Natural Science Museum, Ulyanovsk State University, Ulyanovsk, Russia; 7 Lomonosov Moscow State University, Leninskie Gory 1, GSP-1, Moscow 119991, Russia

**Keywords:** Plesiosauria, Pliosauridae, Cretaceous, macrophagy, convergence, *Makhaira rossica*

## Abstract

During the Middle and Late Jurassic, pliosaurid plesiosaurs evolved gigantic body size and a series of craniodental adaptations that have been linked to the occupation of an apex predator niche. Cretaceous pliosaurids (i.e. Brachaucheninae) depart from this morphology, being slightly smaller and lacking the macrophagous adaptations seen in earlier forms. However, the fossil record of Early Cretaceous pliosaurids is poor, concealing the evolution and ecological diversity of the group. Here, we report a new pliosaurid from the Late Hauterivian (Early Cretaceous) of Russia. Phylogenetic analyses using reduced consensus methods recover it as the basalmost brachauchenine. This pliosaurid is smaller than other derived pliosaurids, has tooth alveoli clustered in pairs and possesses trihedral teeth with complex serrated carinae. Maximum-likelihood ancestral state reconstruction suggests early brachauchenines retained trihedral teeth from their ancestors, but modified this feature in a unique way, convergent with macrophagous archosaurs or sphenacodontoids. Our findings indicate that Early Cretaceous marine reptile teeth with serrated carinae cannot be unequivocally assigned to metriorhynchoid crocodylomorphs. Furthermore, they extend the known diversity of dental adaptations seen in Sauropterygia, the longest lived clade of marine tetrapods.

## 1. Introduction

Pliosaurid plesiosaurs appear in the fossil record by the earliest Jurassic as small, longirostrine taxa [1–3] and became gigantic apex predators during the Middle–Late Jurassic, presumably filling the vacated niche of rhomaleosaurid plesiosaurs [2] and early neoichthyosaurians like *Temnodontosaurus* [4]. Late Jurassic pliosaurids primarily represent the genus *Pliosaurus* and were among the largest plesiosaurians, attaining maximum body lengths estimated around 10–12 m [3,5,6]. They possessed a highly anisodont dentition, including canniniform regions of the premaxilla and maxilla, and composed of ‘trihedral’ or ‘subtrihedral’ teeth bearing one to three smooth or finely crenulated cutting edges (carinae) [3,5,7–13]. These dental features are functionally correlated with macropredaceous habits [11], and differ from those of all other plesiosaurians, including Middle Jurassic pliosaurids [2,7,14], which possess conical teeth lacking well-defined carinae.

The Jurassic–Cretaceous transition had disparate effects on marine reptiles [7,15–17] but strongly affected pliosaurids. So far, just a single lineage has been demonstrated as crossing the Jurassic–Cretaceous boundary, giving rise to the Cretaceous clade Brachaucheninae [7]. Nevertheless, poor sampling of Early Cretaceous marine tetrapods, especially those of the Berriasian–Hauterivian obscures the tempo of Jurassic/Cretaceous turnover. Where fossils of this age are available, they have indicated a transitional fauna comprising the earliest members of Cretaceous groups, alongside representatives of clades that are common in the Late Jurassic, such as cryptoclidid plesiosaurians [7,18], metriorhynchoid thalattosuchians and ichthyosaurs [15,16,19].

Compared to the gigantic macropredatory pliosaurids of the Late Jurassic, brachauchenines are characterized by a number of morphological features suggesting a diet composed of smaller prey items [20]. These include the presence of conical and relatively isodont teeth lacking carinae, proportional elongation of the snout, reduced length of the temporal fenestra and parietal crest, reduction of the ‘crocodile-like’ rostral constriction at the anterior premaxilla/maxilla contact and shorter parietal crest [3,20–22]. However, almost nothing is known about pliosaurids from earlier in the Cretaceous, so their diversity and ecology are basically unknown, with the exception of two partial skeletons with poorly preserved teeth from the Barremian [23] and Aptian [24] of Columbia. We describe a fragmentary pliosaurid from the lower Hauterivian of western Russia (YKM 68249/1-10) that temporally and morphologically bisects the long branch leading to conventional brachauchenines. This pliosaurid shares several brachauchenine synapomorphies, but also bears strongly carinated, trihedral teeth with complex serrations.

## 2. Material and methods

### 2.1 Institutional abbreviations

**ULg**: Université de Liège, Collections de paléontologie animale, Liège, Belgium. **YKM**: Ulyanovskii Oblastnoi Kraevedcheskii Musei I.A. Goncharova (Ulyanovsk Regional Museum of Local Lore named after I.A. Goncharov), Ulyanovsk, Ulyanovsk Region, Russia.

### 2.2 Phylogenetic analysis

We coded YKM 68249/1-10 in the dataset of Benson *et al.* [3], also incorporating ‘*Brachauchenius*’ sp. from Villa Leyva, Colombia [23] and a revised coding of *Anguanax zignoi* [25] (see the electronic supplementary material). Our revision of *Anguanax* resulted in the modification of the scores of 10 characters and in the scoring of nine additional characters that were scored as missing data in previous work [25]. The number of revised scorings (i.e. 19) is large compared with the number of characters that were originally scored in *Anguanax* (i.e. 29) and call into question the level of support for the hypothesis that high evolutionary rates are required to explain its morphology, as suggested by previous work [25].

YKM 68249/1-10 can only be coded for 6% of characters, so its phylogenetic position should be regarded as tentative. We used TNT’s new Technology search (10 random seeds; 100 ratchet iterations, drift and tree fusing activated) to recover most parsimonious trees that were used as a basis for a heuristic search using tree bisection and reconnection (TBR) branch swapping. We performed another analysis pruning unstable taxa identified by the script of Pol & Escapa [26]. The following taxa were identified as unstable and pruned from the second analysis in order to build the reduced consensus: *Pistosaurus* skull, *Pliosaurus brachyspondylus* (CAMSM), *Pliosaurus rossicus*, *Pliosaurus* *irgisensis*, *Kronosaurus queenslandicus* (MCZ 1285), QM F51291, *Eopleiosaurus antiquior*, *Eromangasaurus australis*, and ‘*Brachauchenius*’ sp. Villa Leyva.

### 2.3 Ancestral state reconstruction

We estimated the ancestral state of the character 139, related to cross-sectional shape of the teeth, using maximum-parsimony and maximum-likelihood methods. The maximum-parsimony reconstruction was computed in Mesquite v. 3.04 [27] on the full and reduced strict consensus trees. The maximum-likelihood reconstruction was computed in R [28] using Claddis v. 0.1 [29]. As this method requires a fully resolved tree with branches of positive length, we used the most parsimonious tree with the best stratigraphic fit (RCI) and forced a minimum branch length of 1, using Paleotree v. 2.4 [30] and Strap v. 1.4 [31]).

## 3. Systematic description

Plesiosauria Blainville, 1835 [32]

Pliosauridae Seeley, 1874 [33]

Thalassophonea Benson & Druckenmiller, 2014 [7]

*Makhaira* *rossica* gen. et sp. nov.

LSIDs: urn:lsid:zoobank.org:pub:2C95C409-72C0-45FE-BF58-608657D5382F (Publication); urn:lsid:zoob- ank.org:act:F19A595F-D739-4361-9088-84B7B947DC93 (*Makhaira*); urn:lsid:zoobank.org:act:258CFACB-27D3-44CF-8B04-A12FDDECA55C (*Makhaira rossica*)

(Figures 2–4.)

### 3.1 Holotype, horizon and locality

**Figure 1.**
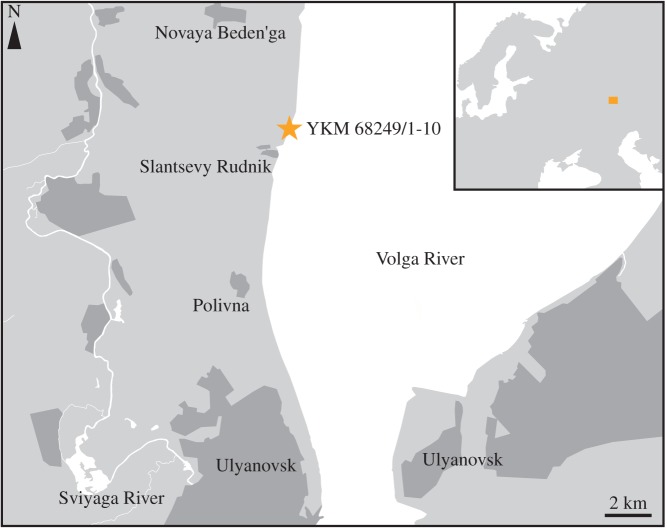
Localization of the section where YKM 68249/1-10 is derived. YKM 68249/1-10 was found in a large pyrite nodule; its precise position within the section (which only contains the *Speetoniceras versicolor* Zone) is unknown.

YKM 68249/1-10, a slightly immature fragmentary skeleton consisting of a partial right premaxilla, the anterior part of the mandible, several teeth, three dorsal vertebrae in anatomical connection, a partial left ischium and a partial right ilium. It is preserved in three dimensions in a series of pyritic limestone nodules found along the banks of the Volga River, 600 m to the north of Slantsevy Rudnik, Ulyanovsk Oblast, Russian Federation (figure 1). The precise level within the section is unknown, but the section only contains Upper Hauterivian (Lower Cretaceous) strata of the *Speetoniceras versicolor* Zone in this locality.

### 3.2 Etymology

From Latinized Ancient Greek ‘μάχαɩρα’ (mákhaira): a blade with a curved outline and Latin ‘rossica’: Russian.

### 3.3 Diagnosis

Pliosaurid plesiosaur characterized by the following autapomorphic features: serrated carinae in which the heights of the denticles are greater than their widths and vary in an undulose (i.e. wave-like) fashion across the apicobasal length of the carina; clustering of the mesial alveoli of the dentary into pairs.

*Makhaira rossica* is also characterized by the following combination of features: procumbent 1st alveolus in premaxilla; 1st alveolus is placed directly anterior to the 2nd alveolus (both shared with a distinct new taxon from the Hauterivian of Russia; V. Fischer 2015, personal observation); long symphysis including more than 10 alveoli (unlike in *Simolestes vorax*, *Liopleurodon ferox*, *Pliosaurus rossicus*, *Pliosaurus macromerus*, *Pliosaurus patagonicus*, *Megacephalosaurus eulerti*, *Brachauchenius lucasi*, *Kronosaurus queenslandicus* [9,20,21,34–36]) absence of symphysial ventral keel (as in *Brachauchenius lucasi*,*Marmornectes candrewi* and rare instances in the Late Jurassic [2,37,38]), streamlined rostrum with no lateral expansion (as in *Hauffiosaurus*spp., *Marmornectes candrewi* and brachauchenines [2,21,22,37,39–41]), trihedral teeth (as in *Pliosaurus* spp. except *P*. *kevani* and *P. andrewsi* [3]), low neural arch (unlike in *Brachauchenius lucasi* [7]).

### 3.4 Description of holotypic cranial remains

The dentigerous portion of the right premaxilla is preserved. However, it is heavily encrusted in pyrite so that not all details are evident. In total, six premaxillary alveoli are present, as in many pliosaurids (e.g. *Marmornectes candrewi*, *Peloneustes philarchus*, *Pliosaurus carpenteri*, ‘*Pliosaurus*’*andrewsi* [2,3,14]). The anteriormost part of the right premaxilla is clearly visible. The anteriormost alveolus is procumbent, facing directly anteriorly from the anterior surface of the snout. All other pliosaurids reported so far have anterior alveoli that face ventrally or weakly anteroventrally [2,3,14,37,39,42,43]. However, we do not recognize this feature as autapomorphic as it is present, albeit differently expressed, in an unpublished specimen that represents a distinct species from the Hauterivian of Russia (V. Fischer July 2015, personal observation). The basal diameter of the 1st crown in YKM 68249/1-10 (*diameter*=10 mm) is slightly reduced compared to more posterior teeth (third *crown*=14 mm). More basal plesiosaurians, including Middle Jurassic thalassophoneans such as *Peloneustes philarchus*, *Simolestes vorax* and *Liopleurodon ferox* have unreduced anteriormost alveolus while *Pliosaurus*, brachauchenines and many plesiosauroids have a strongly reduced anteriormost alveolus [14,43].

The mediolateral width of the preserved portion of the premaxilla is approximately constant, expanding to a maximum of 37 mm in the region of the third-fourth alveoli, and tapering anteriorly and posteriorly from there. Overall, the morphology is slender, lacking significant mediolateral expansion, and lacking the ‘rostral constriction’ that is present around the anterior end of the suture between the premaxilla and maxilla in many Jurassic pliosaurids, including *Peloneustes philarchus* [14], *Simolestes*, *Liopleurodon* [43] and some species of *Pliosaurus* [3,5,10]. This unexpanded morphology is more similar to early-diverging pliosaurids such as *Hauffiosaurus* and *Marmornectes candrewi* [2,40,41], and to other brachauchenines [21,22,37,39].

**Figure 2.**
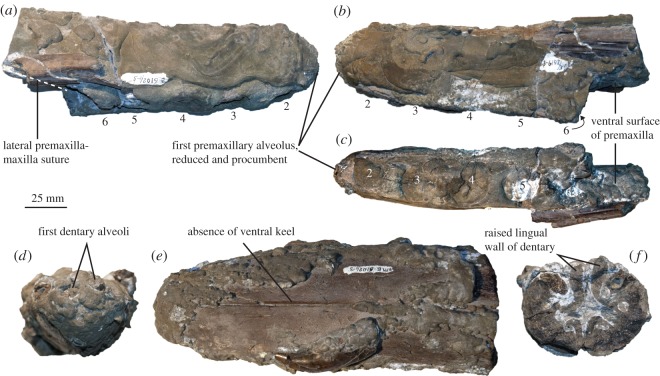
Rostrum of YKM 68249/1-10. (*a*–*c*) Right premaxilla, in (*a*) lateral, (*b*) medial and (*c*) ventral views. Numbers indicate the position of each alveolus. The ventral premaxilla–maxilla suture is located at the 6th alveolus. Note the procumbent 1st alveolus. (*d*–*e*) Anterior part of the symphysis, in (*d*) anterior, (*e*) ventral and (*f*) posterior views.

**Figure 3.**
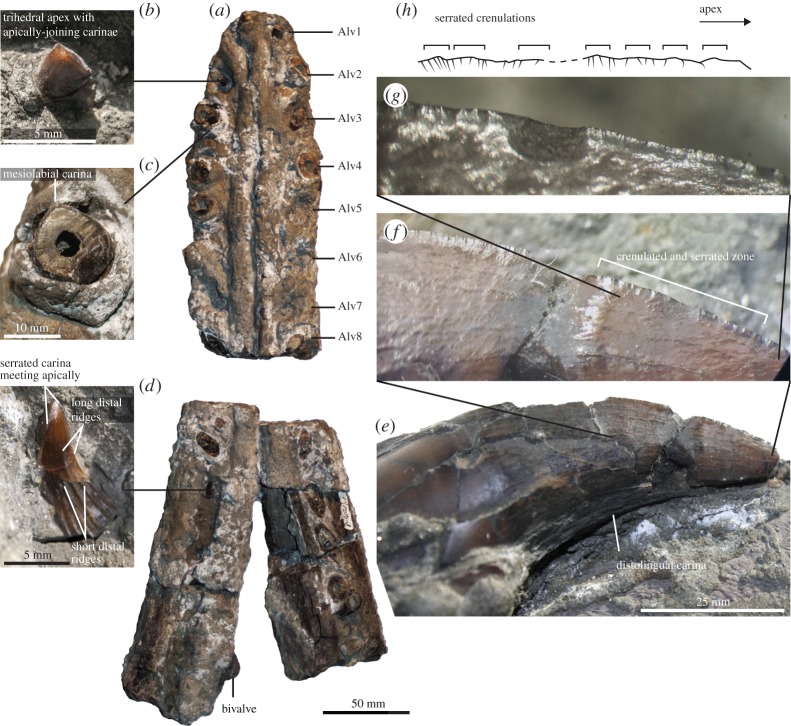
Dentition and mandible of YKM 68249/1-10. (*a*) Mandible in dorsal view. (*b*) Replacement tooth in the 2nd alveolus, showing the trihedral cross section. (*c*) Base of the 3rd alveolus crown, showing the marked mesiolabial carina (the crown fragment has been glued slightly off its original position). (*d*) 1st or 2nd post-symphysis replacement tooth, showing distal ridges and serrated carinae. (*e*,*f*) Successive zooms of the mesiolabial carinae of a broken off crown lying on the ventral surface of the symphysis (figure 2). (*h*) Schematic diagram of the carination, drawn from (*f*). Note the serrated crenulations.

The anterior part of the dentary of YKM 68249/1-10 is preserved in two blocks accounting for most of the symphysis (figures 2 and 3). The symphyseal portion of the dentary is dorsoventrally depressed (height/width at the level of the third alveoli is 0.76) and lacks significant mediolateral expansion, as in the premaxilla, as in *Pliosaurus patagonicus* [36]. A series of small, slit-like replacement alveoli are present ventromedially, along the symphysis. Medial to these, the lingual wall of the dentary forms a dorsally swollen, anteroposteriorly oriented ridge immediately lateral to the symphysis. At the posterior end of the second alveolus, both lingual walls fuse to the form a single raised ridge that extends to the anteriormost alveolus. In many, and perhaps all, plesiosaurians, an oblique groove is present on the lingual wall of each dentary anteriorly [44], and in taxa with long symphyses, such as pliosaurids and polycotylids, these are incorporated into the symphysis [2,3,21]. The locations of the grooves on each side are often asymmetrical [2], and they have sometimes been misinterpreted as sutures [45,46], or jointly described as a ‘heart-shaped depression’ [10,47]. Encrusting pyrite obscures the positions of these grooves in YKM 68249/1-10. However, the groove of the right side seems to be located medial to the fourth alveolus, and that of the left side medial to the posterior part of the third alveolus.

The dorsal exposure of the mandibular symphysial suture is gently sinuous, and the ventral exposure is straight. The symphysis lacks a ventral keel, as in *Brachauchenius lucasi*,*Marmornectes candrewi* and rare instances in the Late Jurassic [2,37,38]. Most other pliosaurids, however, possess a keel reinforcing the symphysis ventrally [10,14,36]. The break between the two preserved portions of the dentary shows that the Meckelian canal is located at approximately mid height of the dentary.

Eight symphysial alveoli are present in the anterior fragment of the dentary and two additional ones are present in the posterior fragment. A portion is missing between these preserved fragments, and we estimate that the posterior end of the symphysis was located at least 10 cm posterior to the eighth alveolus. Therefore, more than 10 symphysial teeth were originally present, and YKM 68249/1-10 should thus be considered as having an intermediate to long symphysis. The spaces between symphysial alveoli are small, less than half of the alveolar diameter, unlike those pliosaurids with wide interdental spaces, such as *Marmornectes candrewi* [2]. Nevertheless, the interalveolar spaces of YKM 68249/1-10 are distinctly larger than those of most other pliosaurids, which have closely spaced alveoli [7]. Some clustering of successive alveoli into pairs is also present within the anterior part of the dentary.

The splenial participates ventromedially in the symphysis. It is present as a narrow, 1.5 mm wide ventral exposure at the posterior end of the anterior fragment, tapering anteriorly to a point. The ventral exposure of the splenial at the anterior end of the posterior fragment is 7 mm wide, suggesting that only a small portion of the mandible is missing (we estimate 3–4 cm), as also evidenced by the general shape of the dentary. The ventral surface of the splenial is flat and there is an approximately 90^°^ angle between its ventral and medial surfaces. Posterior to the symphysis, the splenial thickens dorsoventrally to become a sheet-like bone that covers the ventral portion of the medial surface of the dentary. Pyrite covering of the dorsal portion of the mandible makes it difficult to determine whether the coronoid contributed to the symphysis, as it does in other pliosaurids. The anteriormost part of the angular is seen in ventral view between the splenial and dentary, tapering anteriorly.

Teeth are large and lingually recurved. They vary slightly in size along the anterior part of the mandible: most symphysial teeth have a basal crown diameter of 14 mm, reaching 16 mm in the fourth right symphysial tooth and the isolated tooth. By contrast, the fifth tooth posterior to the symphysis has a basal crown diameter of 12 mm. This essentially isodont condition differs markedly from many Jurassic pliosaurs that have anisodont dentitions, in which a strong difference of tooth size is present between symphysial teeth and those just posterior to the symphysis [35]. Anisodont dentitions even occur in some longirostrine taxa, such as *Peloneustes philarchus* [35], although others, such as *Marmornectes candrewi* and *Hauffiosaurus* spp., have more isodont teeth [2,41]. The anteriormost dentary tooth of YKM 68249/1-10 is slightly smaller than the others, with a preserved diameter of 10 mm and is slightly procumbent.

All teeth of YKM 68249/1-10 appear trihedral apically, with a prominent and acute mesiolabial carina (C1) that reaches the base of the crown. Another, less prominent carina (C2) is present along the distolingual surface. Basally, the mesial and labial surfaces appear flattened, while the rest of the tooth is rounded in cross section. The preserved apices (from the large disarticulated tooth, the second left replacement tooth and the first left replacement tooth posterior to the symphysis) have a markedly triangular cross section, with three carinae. This indicates that a third, distolabial, carina (C3) is present, but only apically. The crown is thus subtrihedral [3,12,35] basally and trihedral (or cross-sectionally ‘subtriangular’ [3]) apically. This morphology is also found in the slightly reduced anteriormost right dentary tooth. Trihedral teeth are common among Late Jurassic pliosaurids, especially in the genus *Pliosaurus* [5,10,12,13,36,48,49], and subtrihedral teeth are also present in specimens of this age [3]. A single isolated trihedral tooth was reported by Zverkov [50], from the Valanginian of Russia. However, plesiosaurians with dental cutting edges, or carinae, have not otherwise been reported from the Cretaceous until now.

At least two of the three carinae (C1 and C3) are textured by complex serrations. In addition to being slightly scalloped, the C1 carina of YKM 68249/1-10 is wave-like apically, and the tooth surfaces bear a series of fine ribs perpendicular to the carina (figure 3). Fine and smooth longitudinal ridges texture the distal and lingual surfaces of the replacement crowns, suggesting that some of all ridges disappear during dental ontogeny. Four ridges are interrupted 7 mm basally to the apex, while two continue up to 3 mm before the apex. Serration of the carinae is present in Jurassic trihedral pliosaurid teeth (e.g. BRSMG Cd6172, *Pliosaurus carpenteri*; BRSMG Cc332, *Pliosaurus westburyensis*), although it has not generally been mentioned in the literature [12,13,49,51]. However, the denticles of Jurassic pliosaurids have apicobasal lengths that are similar to their length projecting from the tooth, whereas those of YKM 68249/1-10 project further from the tooth than their apicobasal length. Furthermore, we have not observed the wave-like morphology of the carina of YKM 68249/1-10 in any other pliosaurid. *Makhaira rossica* thus exhibit ziphodonty (either true or false; see [52–54] for definitions of these terms). The type of ziphodonty can only be verified by making destructive cross sections, which were not attempted because of the limited sample of large functional tooth available in the holotype.

### 3.5 Description of holotypic postcranial remains

**Figure 4.**
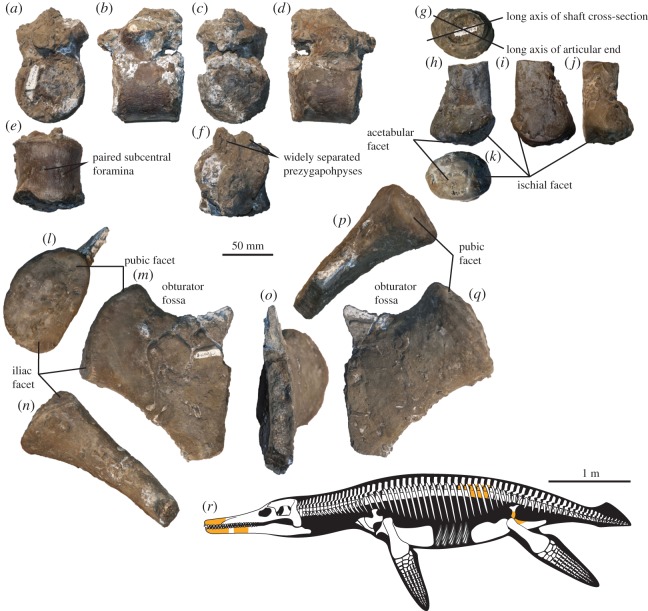
Postcranial remains of YKM 68249/1-10. (*a*–*f*) Dorsal centrum, in (*a*) anterior, (*b*) right lateral, (*c*) posterior, (*d*) left lateral, (*e*) ventral and (*f*) dorsal views. (*g*–*k*) Right ilium in (*g*) dorsal, (*h*) medial, (*i*) lateral, (*j*) posterior and (*k*) ventral views. (*l*–*q*) Left ischium in (*l*) anterolateral (glenoid), (*m*) dorsal, (*n*) posterolateral, (*o*) medial, (*p*) anteromedial and (*q*) ventral views. (*r*) Reconstruction of *Makhaira rossica* based on Late Jurassic pliosaurids and mid-Cretaceos brachauchenines; the orange coloured parts indicate fossils preserved in YKM 68249/1-10.

Two dorsal neural arches are preserved without their neural spines and are extensively covered by pyrite. The articular facet with the centrum is gently convex in lateral view. The zygapophyses are flat and planar and their combined width is much narrower than the centrum width (40 versus 69 mm; figure 4). The neural arch is low, unlike in *Brachauchenius lucasi* [7]. All neural arches appear firmly attached to their corresponding centrum, but a suture is still visible, at least laterally, suggesting this specimen has not yet reached osteological maturity, although even some very large specimens of the pliosaurid *Pliosaurus* show evidence of features usually linked to osteological immaturity [3].

Three dorsal vertebrae are preserved in articulation. These centra are moderately elongated, with a length/height ratio around 1. The centra are slightly wider mediolaterally than high dorsoventrally, with a width/height ratio just less than 1.1. Paired subcentral foramina, are present, but poorly preserved, and an additional nutrient foramen is also visible on each lateral surface of the centrum, where it is not encrusted by pyrite. The ventral surface of the centrum is mediolaterally convex, and no lateral or ventral keel is present; the peripheral surface of the centra is smooth and gently concave.

The medial part of the left ischium is preserved. Its dorsal surface is flat near the acetabular facet and otherwise slightly concave. The ventral surface is slightly saddle-shaped. The posterolateral margin is thick, rounded and straight while the anterior edge forming the anterior part of the obturator fossa is thin and strongly concave in dorsal view.

The articular part of the right ilium is preserved. It bears a large, ventrally facing facet, and a smaller, ventromedially facing facet. The smaller facet is identified as the ischial facet, by comparison with other thalassophoneans, and is semioval. The larger facet is the iliac portion of the acetabulum and has a rounded outline. The shaft of the ilium has a suboval cross section that is longer anteroposteriorly than mediolaterally (41×32 mm). The posterolateral surface of the ilium is approximately flat, whereas the anteromedial surface is more strongly concave anteroposteriorly. The long axis of the cross section of the shaft forms an angle of approximately 40^°^ with the long axis of the acetabular end.

## 4. Results

The phylogenetic analysis of the full dataset recovered more than 20 000 trees of 1345 steps. The topology of the strict consensus tree is similar to previous analyses using this dataset, except that thalassophoneans more derived than *Liopleurodon ferox* form a large polytomy; one exception is a clade containing *Pliosaurus kevani* + *Pliosaurus* cf. *kevani* + *Pliosaurus carpenteri* + *Pliosaurus funkei*. The pruned analysis recovered 780 most parsimonious trees of 1317 steps. In the strict consensus tree, Thalassophonea is better resolved, forming two derived clades closely related to *Gallardosaurus iturraldei*: Brachaucheninae and *Pliosaurus* spp. The resolution of Brachaucheninae is incomplete but stratigraphically congruent: *Makhaira rossica* is recovered as the most basal brachauchenine, being the sister taxon of a clade of derived brachauchenines from the Cenomanian-Turonian: *Brachauchenius lucasi*, (holotype and MNA V9433), *Megacephalosaurus eulerti* and *Polyptychodon* sp. (DOKDM) [3,21,55,56]. Consequently, we interpret *Makhaira rossica* as an early brachaucheninine. Features unambiguously supporting *Makhaira rossica* + Cenomanian-Turonian pliosaurs in the strict reduced consensus tree are: character 1.0 (no transverse constriction of the rostrum at the premaxilla–maxilla suture), character 113.0 (anteriorly tapering mandibular symphysis in ventral view) and character 114.0 (no ventral keel on the symphysis).

**Figure 5.**
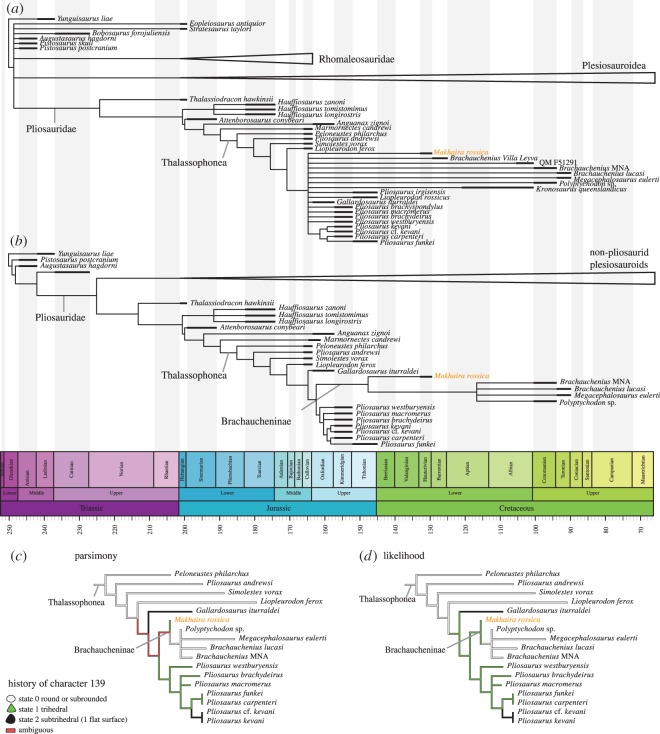
Phylogenetic position of *Makhaira rossica* and ancestral state reconstructions of character 139, related to crown shape. (*a*) Strict consensus of the maximum-parsimony analysis of the full dataset. (*b*) Strict consensus of the maximum-parsimony analysis of the reduced dataset. (*c*) Results of maximum-parsimony method for ancestral state reconstruction (using Mesquite [27]). (*d*) Results of likelihood method for ancestral state reconstruction (using Claddis [29]).

Parsimonious reconstruction of ancestral states recovered equivocal ancestral states for this character 139, describing the dental morphology, in taxa more derived than *Liopleurodon ferox* (figure 5). Maximum-likelihood ancestral state reconstruction supports the ancestrality of trihedral teeth for all thalassophoneans more derived then *Liopleurodon ferox*, and thus subsequently lost in *Pliosaurus kevani* (state 2, subtrihedral) and brachauchenines more derived than *Makhaira rossica* (state 0, conical).

## 5. Discussion and conclusion

### 5.1 Ecology of Early Cretaceous pliosaurids

*Makhaira rossica* shares morphological features with both Late Jurassic and Mid-Cretaceous pliosaurids, detailing the tempo of morphological evolution in the early history of Brachaucheninae. Osteological features often associated with macrophagy, and widely present in Middle–Late Jurassic pliosaurids such as the spatulate rostrum and the expanded caniniform teeth were seemingly lost early in the evolution of brachauchenines. However, the incompletely resolved phylogenetic position of *Makhaira rossica* within Brachaucheninae, and the presence of these features in some other Cretaceous pliosaurid specimens whose phylogenetic affinities were not resolved by our analysis (e.g. [24,39]) raises a number of questions regarding the evolution and biodiversity of early members of that clade. Specifically, it seems that Early Cretaceous pliosaurids exhibit multiple ecomorphologies that are in need of further study. Because of the poor record of Early Cretaceous pliosaurids, it is still unclear whether trihedral, strongly carinated teeth constitute the ancestral condition of derived thalassophoneans or were acquired convergently in *Makhaira rossica*, *Pliosaurus* and currently enigmatic taxa such as ‘*Pliosaurus*’ *rossicus*. Parsimony-based methods are ambiguous while likelihood methods suggest that trihedral teeth are a synapomorphy of *Pliosaurus* + Brachaucheninae, that was subsequently lost within Brachaucheninae. In this scenario, *Makhaira rossica* thus retained the ancestral state of that trait, but modified it via a unique serration pattern.

*Makhaira rossica* departs from both Late Jurassic and Cretaceous thalassophoneans by its smaller size: the largest dorsal centrum is 72 mm wide. Nevertheless, fusion of neurocentral suture suggests osteological maturity for this specimen [57]. For comparison, the last cervical centrum of the late Barremian ‘*Brachauchenius*’ sp. is 117 mm wide, the largest dorsal centrum of *Brachauchenius lucasi* is 90 mm wide and the width of those of *Kronosaurus* *queenslandicus* and ‘*Kronosaurus*’ *boyacensis* exceed 150 mm and 170 mm, respectively [20,23,24]. *Makhaira rossica* markedly differs from Cretaceous thalassophoneans by having relatively large teeth and dental adaptations reminiscent of macrophagous predators such as theropod dinosaurs [58] or thalattosuchians crocodyliforms [54]. Unexpectedly, because of their densely serrated and wave-like pattern, the carinae of YKM 68249/1-10 appear larger and more complex than in other macrophagous marine tetrapods such as *Mosasaurus hoffmani* (V. Fischer 2015, personal observation on ULg PA.25119), *Dakosaurus* *maximus* ([54]; V. Fischer 2015, personal observation on ULg PA.6600) or *Geosaurus*, the latter being regarded as having ‘hypercarnivorous’ adaptations [54]. *Makhaira rossica* is also unique among plesiosaurs in having trihedral but moderately widely spaced teeth. Contrary to carination and serration, previous authors have not generally assigned a specific functional interpretation to the presence of wide interalveolar spacing. However, we note that the carinated teeth of macrophagous marine reptiles are usually closely spaced [11,59].

*Makhaira rossica* thus indicates that pliosaurids explored previously unrecognized niches during the Early Cretaceous, with the presence of a smaller bodied taxon possessing clear yet distinctive macrophagous adaptations. By being the first sauropterygian to develop complex serration of its carinae, *Makhaira rossica* further exemplifies the profound diet-driven morphofunctional convergences that evolved among Mesozoic marine reptiles [11,60].

### 5.2 Implications for metriorhynchid extinction

An isolated crown from the Aptian of Sicily (MSNC 4475) has been recently regarded as evidence for the late survival of geosaurine metriorhynchid crocodyliforms, several million years after their supposed extinction [61]. However, although they do not yet co-occur within a single pliosaurid taxon, all the features of MSNC 4475 described in [61] can now be shown to have been present among Cretaceous pliosaurids (‘The conical shape of the tooth crown, noticeable lingual curvature, presence of mesial and distal carinae, and microscopic denticles along the carinae’ [61], p. 610). We also note that MSNC 4475 appears weakly trihedral in apical view ([61]; figure 2*f*). Moreover, fine, smooth and widely spaced apicobasal ridges restricted to one surface of the tooth and the triangular or approximately triangular cross section of the crown are other features shared between MSNC 4475 and *Makhaira rossica*. Differences between these two specimens are also present: the apicobasal ridges are not located on the curved side in the large tooth of *Makhaira rossica* (but such ridges are present in one small replacement tooth (figure 3) and thus possibly variable with dental development in *Makhaira rossica*), and the weak development of a trihedral cross section in the Sicilian tooth. It is not currently possible to make a definitive statement on the affinities of MSNC 4475, which clearly is an important specimen and potentially illustrates the profound convergence of *Makhaira rossica* with macrophagous archosaurs. However, future discoveries are likely to clarify whether MSNC 4475 is a late-surviving, low-latitude metriorhynchid or a brachauchenine pliosaurid.

## Supplementary Material

Supplementary material. Includes: pictures of a bivalve associated with YKM 68249/1-10, stratigraphic age data for all taxon incorporated in the phylogeny, revised phylogenetic coding for Anguanax zignoi, additional figures of strict consensus topologies, detailed results from the ancestral state reconstruction and R scripts. -Nexus files. With character-taxon matrices and most parsimonious trees.

## Supplementary Material

Plios_full_BL.nex

## Supplementary Material

Plios_red_BL.nex

## Data accessibility

Additional data are uploaded in the electronic supplementary material; we included pictures of a bivalve associated with YKM 68249/1-10, stratigraphic age data for all taxon incorporated in the phylogeny, revised phylogenetic coding for *Anguanax zignoi*, additional figures of strict consensus topologies, detailed results from the ancestral state reconstruction and R scripts. Nexus files with character-taxon matrices and most parsimonious trees will be deposited in the DataDryad repository (http://dx.doi.org/10.5061/dryad.1d52k).
