# Supplementary material for: Peculiar macrophagous adaptations in a new Cretaceous pliosaurid
Source: R Soc Open Sci. 2015 Dec 23;2(12):150552. doi: 10.1098/rsos.150552 (PMC4807462; doi:10.1098/rsos.150552)
Supplement: Supplementary material. Includes: pictures of a bivalve associated with YKM 68249/1-10, stratigraphic age data for all taxon incorporated in the phylogeny, revised phylogenetic coding for Anguanax zignoi, additional figures of strict consensus topologies, detailed results from the ancestral state re [file rsos150552supp1.docx]

**SUPPLEMENTARY INFORMATION**

**Peculiar macrophageous adaptations in a new Cretaceous pliosaurid**

Valentin Fischer^1,2^, Maxim S. Arkhangelsky^3,4^, Ilya M. Stenshin^5^, Gleb N. Uspensky^6^, Nikolay G. Zverkov^7^ & Roger B. J. Benson^1^.

^1^Department of Earth Sciences, University of Oxford, OX1 3AN Oxford, UK.

^2^Geology Department, Université de Liège, 14 allée du 6 Août, 4000 Liège, Belgium.

^3^Saratov State Technical University, Politekhnicheskaya Ul. 77, 410054 Saratov, Russia.

^4^Saratov State University, Astrakhanskaya Ul. 83, 410012 Saratov, Russia.

^5^I.A. Goncharov Ulyanovsk Regional Natural History Museum, Boulevard Novyi Venets 3/4, Ulyanovsk, 432000 Russia.

^6^Natural Science Museum, Ulyanovsk State University, Ulyanovsk, Russia.

^7^Lomonosov Moscow State University, Leninskie Gory 1, 119991 GSP-1, Moscow, Russia.

**1. Indeterminate bivalve remains associated with YKM 68249/1-10**


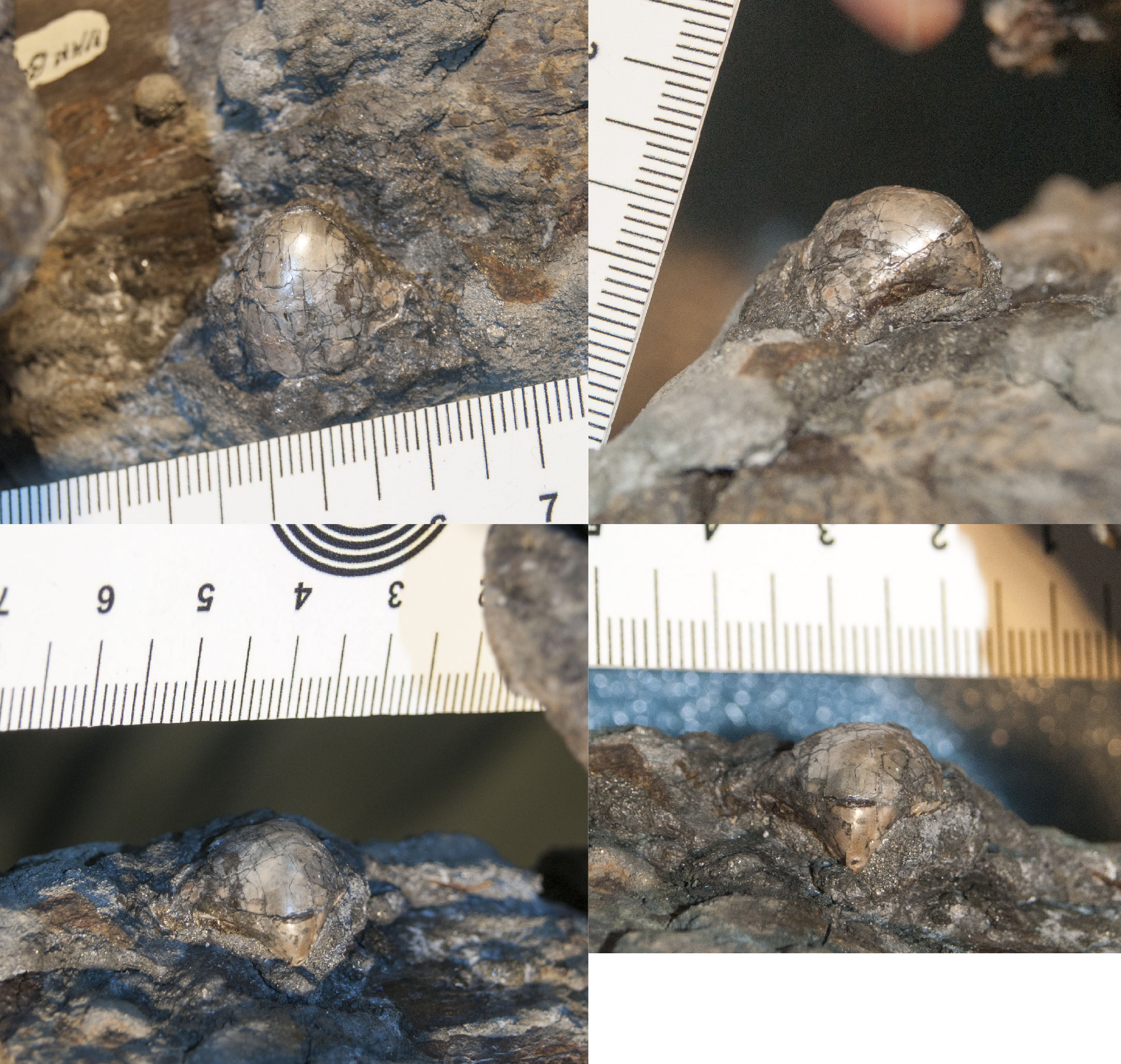


**Fig. S1. Indeterminate bivalve on the medial surface of the left mandibular ramus.**

**2. Selected measurements of YKM 68249/1-10**

| Dorsal centra | Length | Width | Height |
| --- | --- | --- | --- |
| 1 | 65 | 69 | 63 |
| 2 | 68 | 72 | 64 |
| 3 | 61 | 70 | 65 |
|  |  |  |  |
| Ischium | Anterior-posterior length | Height glenoid | Length glenoid |
|  | 111 | 69 | 94 |
|  |  |  |  |
| Crown basal diameters | Left dentary ramus | Right dentary ramus | |
| 1st | 10 | NA |  |
| 2nd | NA | 13 |  |
| 3rd | 14 | NA |  |
| 4th | 14 | 15 |  |
| 5th | 14 | NA |  |
| 6th | 12 | NA |  |
| Isolated large tooth | NA | 15.5 |  |
| 4th post symphysis | NA | 12 |  |
| 5th post symphysis | NA | 12 |  |

**Table S1. Selected measurements of YKM 68249/1-10 (in mm).**

**3. Revised coding for *Aguanax zignoi***

We included *Aguanax zignoi* from the middle Oxfordian of Italy based on the descriptions and figures of Cau & Fanti [1,2]. Cau & Fanti [1] scored 24 characters based on the character list of Benson et al. [3,4]. For each character scored, we conducted a comparative survey of pliosaurids and other plesiosaurians, based on our data and the literature. The objective was to be sure that character states were assigned based on well-supported observations of similarity to other taxa possessing the same character states, and not just on superficial similarity based strictly/solely on the reading of character state descriptions. The resulting revisions are explained below. Our revision of their scores resulted in the modification of ten of their scores and in the scoring of nine additional characters.

Cau & Fanti [1] found high rates of phenotypic character evolution in the terminal branch leading to *Anguanax*. However, our modifications converted several of their scores that were distinct from those pliosaurids most closely related to *Anguanax* to scores that were similar. It is therefore likely that their interpretation of high evolutionary rates in fact results from the quantitative exaggeration of character state dissimilarity between *Anguanax* and its close relatives due to erroneous assignment of character states. The analysis of evolutionary rates based on character/taxon matrices has a long history [5] and is currently undergoing a renaissance [6,7]. However, interpretations of the results of such analyses should be interpreted cautiously as the screening of similarity propositions encoded in these matrices requires considerable expertise and is a time-consuming activity.

Modifications (10)

-Character 4 can be scored as {12} as it is likely that the incompletely preserved rostrum comprised at least 45% of the total skull length. In support of this proposition, we note that Cau & Fanti [2] stated that the specimen could be referred to the a basal longirostrine clade of pliosaurids.

-Character 6 should be scored as 1 rather than 0 due to the presence of a convex projection in the anterodorsal orbital margin that is topologically similar to that present in other thalassophoneans (Cau & Fanti [2], fig. 2; Ketchum & Benson [8]).

-Character 116 should be scored as 1 rather than 0 as the retroarticular process is clearly longer anteroposteriorly than is the glenoid, as in early-diverging, longirostrine pliosaurids such as *Hauffiosaurus* spp., *Marmornectes candrewi* and *Peloneustes philarchus* [8–10].

-Character 137 should be scored as 0 rather than 1 as the enamel ridge figured by Cau & Fanti ([2]: fig. 3A3) is comparable in relative thickness to those seen in other pliosaurids [8,10], and not fine as in some other plesiosaurians (RBJB, pers. obs.; although the ridge in *Anguanax* is figured at very small size).

-We scored character 150 as 1 rather than 0 as the axial neural spine of *Anguanax* is low dorsoventrally. We acknowledge that this character is phrased as distinguishing ‘transversely narrow’ axial neural spines (state 0) from the ‘transversely broad’ axial neural spines seen in some pliosaurids (state 1). However, we view it as being important that the axial neural spine of *Anguanax* is more similar in overall relative dimensions to those of other taxa scored as state 1 (e.g. *Hauffiosaurus*; [9]) than to other taxa (e.g. *Stratesaurus taylori* [11]).

-We scored character 175 as ? rather than 0 as the anteroventral ‘lip’ of thalassophoneans can be a small structure that is difficult to perceive in ventral view, and can be present only in some cervical centra.

-Character 188 is scored as 0 (caudal rib facets located dorsally, contacting or almost contacting neural arch) rather than 1 (placed dorsally, neural arch does not form part of facet). This was difficult to determine objectively as neither description of the specimen [1,2] contains sufficiently large images of the caudal vertebrae. However, Cau & Fanti ([2]: p.647) described the caudal rib facets as being “placed almost entirely on the centra”, suggesting that the neural arch does contact the rib facet.

-Character 207 is scored as {01} rather than 0, indicating uncertainty as to whether the posterolateral cornu of the coracoid extends laterally to the level of the glenoid (state 1), or not (state 0). We list this amendment as being subjective because the scapulocoracoid is crushed and fragmented, and we found it difficult to establish the morphology and orientation based on the information provided by Cau & Fanti [1,2].

-For the same reason, we scored character 210, concerning the orientation of the posterior margin of the coracoid, as ? rather than 3 (posterolaterally oriented).

-Character 270 should be scored as state 1 (phalanges short and robust) rather than state 0 (long and slender). We recognise that state 0 is defined as ~2-3 times as long proximodistally as broad anteroposteriorly, which is accurate for at least some of the preserved phalanges of *Anguanax* (measured from Cau & Fanti [2]: fig. 5: ratios = 2.7, 2.2, 1.93). However, this quantitative definition is in need of revision as the proportions of the preserved phalanges of *Anguanax* are similar to those of *Marmornectes* and other pliosaurids [10,12], which are scored as state 1, and proportionally more robust than in taxa such as *Hauffiosaurus* [9,13], which are scored as state 0.

Added scores (9)

-Character 3 should be scored as 0 rather than ? as the ratio of orbit length to temporal fenestra length is 0.46 (measured from [2]: fig. 2).

-Character 121 should be scored as 0 rather than ? as the lateral surface of the mandible is not concave dorsoventrally.

-Character 144 should be scored as 0 rather than ? as the atlantal centrum is preserved, and lacks lateral processes that could contribute to the lateral surfaces of the atlantal cup ([2]: fig. 3B).

-Character 155 should be scored as 0 rather than ? as the cervical centra lack the ventral notch (= overall ‘binocular’ shape) seen in elasmosaurids [14].

-Character 156 should be scored as 1 rather than ?, as subcentral foramina were described by Cau & Fanti [2] as being present on the ventral surface of the preserved cervical centrum, and state 1 is the only state that includes this reported morphology.

-Character 139 should be scored as 0 rather than ? as the preserved teeth have sub oval cross sections.

-Character 243 is scored as 0 rather than ? as the proximal ends of the tibia and fibula appear not to be convex ([2]: fig. 6), precluding the presence of a tongue-in-groove articulation between epipodials and proposals.

-Character 247 should be scored as 0 rather than ? as the distal end of the femur is uniformly convex, lacking a distinct angle between the epipodial facets.

-Character 248 should be scored as 0 rather than ? as the epipodial facets, visible on the femur, are oriented at an oblique angle to each other.

**4. Character-taxon matrix**

See supplementary nexus files (with most parsimonious trees incorporated) Plios_full_BL.nex and Plios_red_BL.nex

**5. Taxon age data (full dataset; remove the pruned taxa for the reduced dataset)**

FAD LAD

Yunguisaurus_liae 242 237

Pistosaurus_postcranium 247.2 242

Pistosaurus_skull 247.2 242

Augustasaurus_hagdorni 247.2 242

Bobosaurus_forojuliensis 237 227

Macroplata_tenuiceps 201.3 199.3

Anningasaura_lymense 201.3 199.3

Stratesaurus_taylori 201.3 199.3

Avalonnectes_arturi 201.3 199.3

Eurycleidus_arcuatus 201.3 199.3

Meyerasaurus_victor 183 179.3

Maresaurus_coccai 170.3 168.3

Borealonectes_russelli 166.1 163.5

Rhomaleosaurus_megacephalus 201.3 199.3

Archaeonectrus_rostratus 201.3 199.3

Rhomaleosaurus_cramptoni 182.7 174.1

Rhomaleosaurus_zetlandicus 182.7 174.1

Rhomaleosaurus_thorntoni 182.7 174.1

Thalassiodracon_hawkinsii 201.3 199.3

Hauffiosaurus_longirostris 182.7 174.1

Hauffiosaurus_tomistomimus 182.7 174.1

Hauffiosaurus_zanoni 182.7 174.1

Marmornectes_candrewi 166.1 163.5

Peloneustes_philarchus 166.1 163.5

Simolestes_vorax 166.1 163.5

Pliosaurus_funkei 152.1 145

Pliosaurus_westburyensis 157.3 152.1

Pliosaurus_carpenteri 157.3 152.1

Pliosaurus_brachydeirus 157.3 152.1

Pliosaurus_macromerus 157.3 152.1

Pliosaurus_brachyspondylus 157.3 152.1

Pliosaurus_cf_kevani 157.3 152.1

Pliosaurus_kevani 157.3 152.1

Gallardosaurus_iturraldei 163.5 157.3

Pliosaurus_rossicus 152.1 145

Pliosaurus_irgisensis 152.1 145

Pliosaurus_andrewsi 166.1 163.5

Liopleurodon_ferox 166.1 163.5

Kronosaurus_queenslandicus 121 100.5

Polyptychodon_sp 100.5 93.9

Megacephalosaurus_eulerti 93.9 89.8

Brachauchenius_lucasi 93.9 89.8

Brachauchenius_MNA 100.5 93.9

QM_F51291 105.5 100.5

Attenborosaurus_conybeari 199.3 190.8

Plesiosaurus_dolichodeirus 199.3 190.8

Eopleiosaurus_antiquior 201.3 199.3

Eretmosaurus_rugosus 199.3 190.8

Westphaliasaurus_simonsensii 190.8 182.7

Seelyosaurus_guilelmiimperatoris 182.7 174.1

Microcleidus_tournemirensis 182.7 174.1

Microcleidus_brachypterygius 182.7 174.1

Microcleidus_homalospondylus 182.7 174.1

Plesiopterys_wildi 182.7 174.1

Cryptoclidus_eurymerus 166.1 163.5

Tricleidus_seeleyi 166.1 163.5

Muraenosaurus_leedsii 166.1 163.5

Kimmerosaurus_langhami 152.1 145

Pantosaurus_striatus 163.5 157.3

Picrocleidus_beloclis 166.1 163.5

Tatenectes_laramiensis 163.5 157.3

Plesiosaurus_mansellii 157.3 145

Colymbosaurus_trochanterius 157.3 145

Djupedallia_engeri 157.3 145

Spitrasaurus_spp 157.3 145

Abyssosaurus_nataliae 132.9 129.4

Umoonasaurus_demoscyllus 125 100.5

Nichollssaura_borealis 113 110.5

Leptocleidus_capensis 139.8 132.9

Leptocleidus_superstes 129.4 125

Vectocleidus_pastorum 129.4 125

Brancasaurus_brancai 145 139.8

Gronausaurus_wegneri 145 139.8

Speeton_Clay_plesiosaurian 132.9 129.4

Wapuskanectes_betsynichollsae 113 107.8

Futabasaurus_suzukii 86.3 83.6

Callawayasaurus_colombiensis 125 121

Eromangasaurus_australis 105.5 100.5

Kaiwhekea_katiki 72.1 66

Aristonectes_parvidens 83.6 66

Libonectes_morgani 93.6 89.8

Hydrotherosaurus_alexandrae 72.1 66

Edgarosaurus_muddi 105.5 100.5

Plesiopleurodon_wellesi 100.5 93.9

Richmond_pliosaur 113 100.5

Brachauchenius_Villa_Leyva 129.4 125

Anguanax_zignoi 163.5 157.3

YKM_B6102 132.9 129.4

**6. R script**

library(ape)

library(paleotree)

library(strap)

library(xlsx)

library(Claddis) # CHECK if there is a tab/space between taxon names and characters in the nexus AND no dot in OTU names

trees_full<-read.nexus("Plios_full_BL.nex")

consensus_full<-consensus(trees_full)

ages_full<-read.table("ages_full.txt",header=T)

ts_full <- DatePhylo(consensus_full, ages_full, rlen=3, method="equal")

pdf("full_consensus.pdf", width=10, height=7)

geoscalePhylo(ladderize(ts_full,right=T),ages_full,cex.ts=0.5,cex.tip=0.5)

dev.off()

trees_red<-read.nexus("Plios_red_BL.nex")

morpho_red <- ReadMorphNexus("Plios_red_BL.nex")

consensus_red<-consensus(trees_red)

ages_red<-read.table("ages_red.txt",header=T)

ts_red <- DatePhylo(consensus_red, ages_red, rlen=3, method="equal")

#####Strati congruence

X<-StratPhyloCongruence(trees_red, ages_red, hard=FALSE,randomly.sample.ages=TRUE, fix.topology=TRUE, fix.outgroup=TRUE)

X$input.tree.results

X$samp.permutation.results

X$rand.permutation

best.input.RCI <- X$input.trees[[which.max(X$input.tree.results[, "RCI"])]]

best.RCI<-which.max(X$input.tree.results[, "RCI"])

best.input.GER<-X$input.trees[[which.max(X$input.tree.results[, "GER"])]]

best.GER<-which.max(X$input.tree.results[, "GER"])

####Ancestral states

anc.tree <- minBranchLength(trees_red[[best.RCI]],1) #removing 0-length branches with paleotree

anc.tree$root.time <- ts_red$root.time

anc.state <- AncStateEstMatrix(morpho_red, anc.tree, estimate.allchars=TRUE, estimate.tips=FALSE)

anc.tree$node.label <- anc.state[,139]

plot(anc.tree, show.node.label=T)

**7. Additional phylogeny figures**

**Fig. S2. Timescaled strict consensus resulting from the maximum parsimony analysis of the full dataset.**

**Fig. S3. Timescaled strict consensus resulting from the maximum parsimony analysis of the reduced dataset.**

**8. Ancestral character state reconstruction results**

**Fig. S4. Ancestral state reconstruction of character 139 (crown shape) using parsimony in Mesquite** [15]**.**

**Fig. S5. Ancestral state reconstruction of character 139 (crown shape) using likelihood in Claddis** [16] **in R** [17]**.**

**Additional references**

1. Cau, A. & Fanti, F. 2015 High evolutionary rates and the origin of the Rosso Ammonitico Veronese Formation (Middle-Upper Jurassic of Italy) reptiles. *Hist. Biol.* , 1–11. (doi:10.1080/08912963.2015.1073726)

2. Cau, A. & Fanti, F. 2014 A pliosaurid plesiosaurian from the Rosso Ammonitico Veronese Formation of Italy. *Acta Palaeontol. Pol.* **59**, 643–650.

3. Benson, R. B. J., Evans, M., Smith, A. S., Sassoon, J., Moore-Faye, S., Ketchum, H. F. & Forrest, R. 2013 A giant pliosaurid skull from the Late Jurassic of England. *PLoS One* **8**, e65989. (doi:10.1371/journal.pone.0065989)

4. Benson, R. B. J. & Druckenmiller, P. S. 2014 Faunal turnover of marine tetrapods during the Jurassic–Cretaceous transition. *Biol. Rev.* **89**, 1–23. (doi:10.1111/brv.12038)

5. Westoll, T. S. 1949 On the evolution of the Dipnoi. *Genet. Paleontol. Evol.* , 112–184.

6. Brusatte, S. L., Lloyd, G. T., Wang, S. C. & Norell, M. A. 2014 Gradual assembly of avian body plan culminated in rapid rates of evolution across the dinosaur-bird transition. *Curr. Biol.* **24**, 2386–92. (doi:10.1016/j.cub.2014.08.034)

7. Close, R. A., Friedman, M., Lloyd, G. T. & Benson, R. B. J. 2015 Evidence for a Mid-Jurassic Adaptive Radiation in Mammals. *Curr. Biol.* **25**, 1–6. (doi:10.1016/j.cub.2015.06.047)

8. Ketchum, H. F. & Benson, R. B. J. 2011 The cranial anatomy and taxonomy of *Peloneustes philarchus* (Sauropterygia, Pliosauridae) from the Peterborough Member (Callovian, Middle Jurassic) of the United Kingdom. *Palaeontology* **54**, 639–665. (doi:10.1111/j.1475-4983.2011.01050.x)

9. Benson, R. B. J., Ketchum, H. F., Noè, L. F. & Gómez-Pérez, M. 2011 New information on *Hauffiosaurus* (Reptilia, Plesiosauria) based on a new species from the Alumn Shale member (Lower Toarcian: Lower Jurassic) of Yorkshire, UK. *Palaeontology* **54**, 547–571.

10. Ketchum, H. F. & Benson, R. B. J. 2011 A new pliosaurid (Sauropterygia, Plesiosauria) from the Oxford Clay Formation (Middle Jurassic, Callovian) of England: evidence for a gracile, longirostrine grade of Early–Middle Jurassic pliosaurids. *Spec. Pap. Palaeontol.* **86**, 109–129.

11. Benson, R. B. J., Evans, M. & Taylor, M. A. 2015 The anatomy of *Stratesaurus* (Reptilia, Plesiosauria) from the lowermost Jurassic of Somerset, United Kingdom. *J. Vertebr. Paleontol.*

12. Knutsen, E. M., Druckenmiller, P. S. & Hurum, J. 2012 A new species of *Pliosaurus* (Sauropterygia: Plesiosauria) from the Middle Volgian of central Spitsbergen, Norway. *Nor. J. Geol.* **92**, 235–258.

13. Vincent, P. 2011 A re-examination of *Hauffiosaurus zanoni*, a pliosauroid from the Toarcian (Early Jurassic) of Germany. *J. Vertebr. Paleontol.* **31**, 340–351. (doi:10.1080/02724634.2011.550352)

14. Welles, S. P. 1943 Elasmosaurid plesiosaurs with description of new material from California and Colorado. *Mem. Univ. Calif.* **13**, 125–254.

15. Maddison, W. P. & Maddison, D. R. 2011 Mesquite: A modular sytem for evolutionary analysis.

16. Lloyd, G. T. 2015 Package ‘Claddis’. , 1–24.

17. R Core Team 2015 R: A language and environment for statistical computing.
